# Toremifene, a selective estrogen receptor modulator, significantly improved biochemical recurrence in bone metastatic prostate cancer: a randomized controlled phase II a trial

**DOI:** 10.1186/s12885-015-1871-z

**Published:** 2015-11-02

**Authors:** Tetsuya Fujimura, Satoru Takahashi, Haruki Kume, Tomohiko Urano, Kenichi Takayama, Yuta Yamada, Motofumi Suzuki, Hiroshi Fukuhara, Tohru Nakagawa, Satoshi Inoue, Yukio Homma

**Affiliations:** Department of Urology, Graduate School of Medicine, The University of Tokyo, 7-3-1 Hongo, Bunkyo-ku, Tokyo 113-8655 Japan; Department of Urology, Graduate School of Medicine, The Nihon University, Tokyo, Japan; Department of Geriatric Medicine, Graduate School of Medicine, The University of Tokyo, 7-3-1 Hongo, Bunkyo-ku, Tokyo 113-8655 Japan; Department of Anti-Aging Medicine, Graduate School of Medicine, The University of Tokyo, 7-3-1 Hongo, Bunkyo-ku, Tokyo 113-8655 Japan

**Keywords:** Toremifene, Raloxifene, Androgen deprivation therapy, Biochemical recurrence, Prostate cancer

## Abstract

**Backgrounds:**

Durability of androgen-deprivation therapy (ADT) for prostate cancer (PC) is limited. Additional selective estrogen receptor modulators (SERMs) may prolong the durability of ADT, because androgen and estrogen signaling drive PC progression.

**Methods:**

Men with treatment-naïve bone metastatic PC were randomly assigned in 1:1:1 fashion to receive ADT, toremifene 60 mg plus ADT (TOPADT), or raloxifene 60 mg plus ADT (RAPADT). The primary endpoint was the biochemical recurrence (BCR) rate, and secondary endpoints were changes of scores of the visual analogue scale (VAS) and the functional assessment of cancer therapy (FACT).

**Results:**

A total of 15 men, 5 each, were allocated to one of the three treatment arms. The basal serum prostate-specific antigen (PSA) level was 198 ng/mL (median, range; 30–8428). Bone metastases were graded as 1 (*n* = 11), 2 (*n* = 3), and 3 (*n* = 1) by the extent of disease. During the median follow-up period of 1370 days (range; 431–1983), BCR occurred in 3, 0 and 2 men in ADT, TOPADT and RAPADT group, respectively. The 5-year BCR-free rate was 30, 100 and 53 %, in ADT, TOPADT and RAPADT group, respectively (*p* = 0.04, ADT v.s. TOPADT, *p* = 0.48, ADT v.s. RAPADT and *p* = 0.12, TOPADT v.s. RAPADT). Scores of VAS improved in all groups and remained stable throughout the study. This analysis is limited as a preliminary result in a single center.

**Conclusions:**

Toremifene with conventional ADT significantly improved the BCR rate in treatment-naïve bone metastatic PC. Further clinical trials are warranted to confirm the promising clinical efficacy of this combination therapy.

**Trial registration:**

The protocol was registered at the University Hospital Medical Information Network (UMIN ID;0,000,064,000) in Sep 25, 2011.

**Electronic supplementary material:**

The online version of this article (doi:10.1186/s12885-015-1871-z) contains supplementary material, which is available to authorized users.

## Background

Based on the pioneering work by Huggins [1], androgen deprivation therapy (ADT) has been the primary treatment for advanced prostate cancer (PC). Unfortunately, most advanced cases of PC eventually become castration-resistant (CRPC), despite the continued use of ADT [2]. Novel therapies such as docetaxel, enzaltamide, abiraterone, cabazitaxel and sipuleucel-T [2–5] have been developed to treat CRPC. However, the development of agents that inhibit progression to CRPC may represent alternative therapeutic options for PC.

The results of recent studies have revealed growth regulation of PC via steroid nuclear receptors, which included not only the androgen receptor (AR) [6, 7] but also members of the estrogen receptor (ER) family [8, 9]. ERα and ERβcx (ERβ2) in particular have been implicated in PC progression and PC-related mortality, whereas ERβ inhibits tumor growth [8, 9]. In this regard, selective estrogen receptor modulators (SERMs) are expected to change the clinical course of PC. For example, toremifene, an ERα antagonist in the prostate [10], decreased the incidence of PC in men with high-grade prostatic intraepithelial neoplasia (HGPIN) [11, 12]. Furthermoere, raloxifene inhibited androgen-independent PC growth in 5 (28 %) of 13 patients [13]. However, SERMs have not been fully investigated for use in those with treatment-naïve PC. We hypothesized that additional SERMs may prolong the durability of ADT, because androgen and estrogen signaling drive PC progression. In the present study, we conducted a prospective randomized clinical phase IIA trial to investigate the effects of SERMs (toremifene and raloxifene) when added to ADT in treatment-naïve bone metastatic PC.

## Methods

### Participants

The inclusion criteria were men aged ≥20 years if they had histological confirmed adenocarcinoma of the prostate and radiologically proven bone metastasis with performance status 0, and adequate hepatic, hematological and renal function. Patients who had previous ADT or chemotherapy for PC, deep vein thrombosis, pulmonary embolism or antiphospholipid antibody syndrome were excluded. Bisphosphonate, warfarin, phenobarbital, rifampicin, phenitoin, ampicillin or cholestyramine was not allowed during the study.

Extent of diseases (EOD) of bone metastasis was graded by bone scintigraphy using technetium-99 m-methylene diphosphonate as follows: 0, normal or abnormal due to benign bone disease; 1, number of bony metastases <6, each of which was <50 % of the size of a vertebral body (one lesion approximately the size of a vertebral body would be counted as two lesions); 2, number of bone metastases between 6 and 20, size of lesions as previously described; 3, number of metastases ≥20 but less than a “super scan”, and 4, “super scan” or its equivalent, i.e., more than 75 % of the ribs, vertebrae and pelvic bones [14]. The Japan Cancer of the Prostate Risk Assessment (J-CAPRA) score (range; 0–12) was calculated on the basis of GS, PSA levels and clinical stage [15].

The protocol was approved by the ethical committee (Internal Review Board) at the University of Tokyo Hospital in August 2008 (approval number; P2008054) entitled preliminary study of selective estrogen modulators (SERMs) combined with maximum androgen blockade for metastatic prostate cancer (see Additional file 1: Table S1). And the study was also registered at the University Hospital Medical Information Network (UMIN ID; 0000064000). All patients provided written informed consent. An analysis was performed and reported to the Internal Review Board in the University of Tokyo Hospital every year.

### Study design

**Fig. 1:**
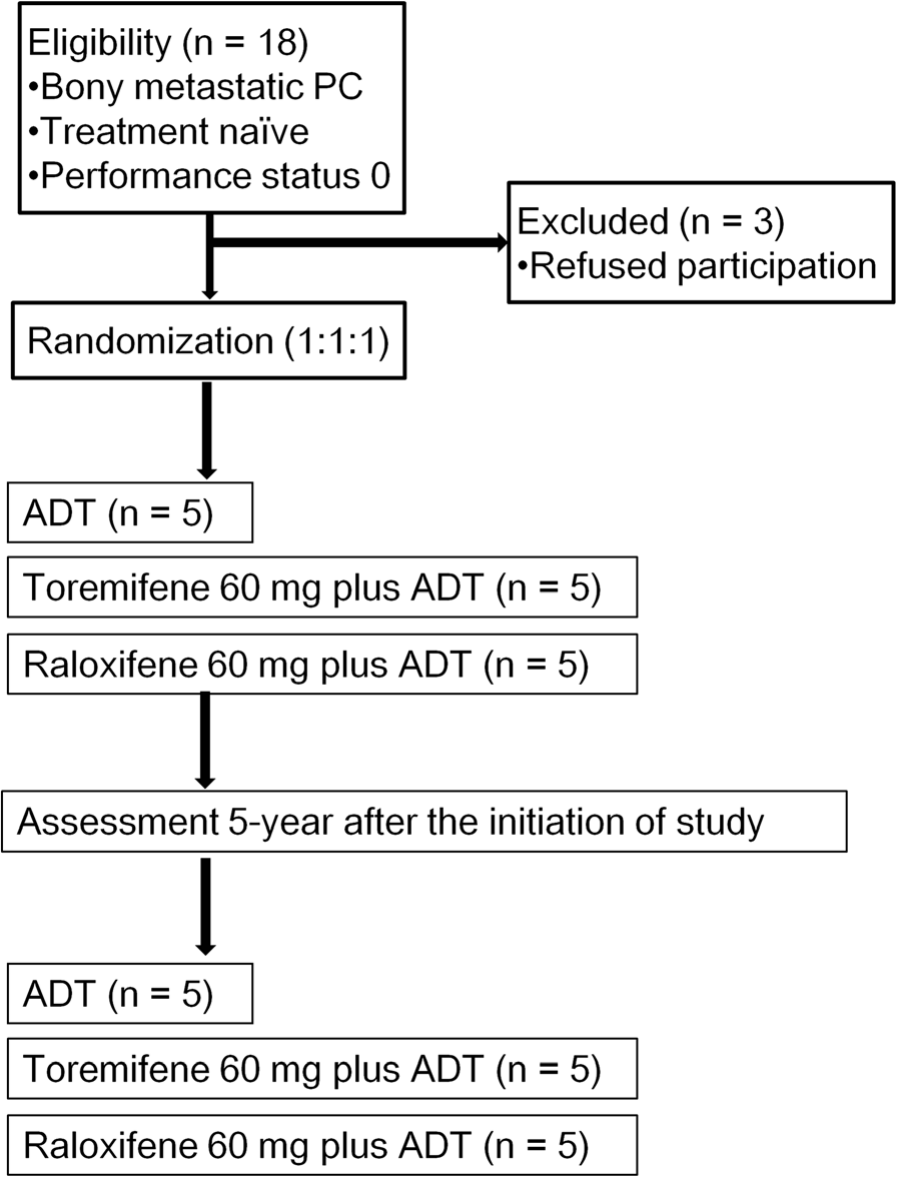
CONSORT flow diagram of recruited patients and follow-up. Men diagnosed with PC by trans-rectal prostate needle biopsy underwent a bone scan. If patients had bone metastasis and agreed to participate in the trial, they were assigned to receive ADT alone, TOPADT or RAPADT in a 1:1:1 ratio (*n* = 15)

Figure 1 shows the consolidated standards of reporting trials (CONSORT) flow diagram of recruited patients and follow-up. Eligible patients were randomly allocated in a 1:1:1 fashion to receive ADT alone, toremifene plus ADT (TOPADT) or raloxifene plus ADT (RAPADT). ADT consisted of castration [bilateral orchiectomy or luteinizing hormone-releasing hormone (LHRH) agonists] combined with 80 mg of bicalutamide. The LHRH agonist was administered throughout, whereas bicalutamide was changed to flutamide on biochemical recurrence after denying anti-androgen withdrawal syndrome. Toremifene (Orion Corporation, Finland) and raloxifene (Eli Lilly Japan K.K.) were given at a dose of 60 mg orally every day combined with aspirin 100 mg daily for prophylactic anti-coagulation.

After PC became hormone-refractory, administration of flutamide was switched to systemic chemotherapy which was performed every 3–4 weeks. If PC became both hormone refractory and chemotherapy-refractory, patients received best supportive care.

### Study end points

Patients were monitored every month during the first year, and every 3 months thereafter. The primary endpoint was the BCR, which was defined as consecutive increase in serum PSA levels to above the patient’s PSA nadir [16]. Secondary endpoints included pain on a visual analogue scale (VAS) and functional assessment of cancer therapy (FACT) in every 3 months [17].

#### Immunohistochemical analysis

The immunohistochemical analyses for AR, ERα and ERβ were performed using the streptavidin-biotin amplification method and an EnVision + visualization kit (Dako, Carpinteria, CA, USA) as previously described [9]. The primary antibody against AR (1:40 dilution), ERα (1:40 dilution) and ERβ (1:200 dilution) was applied and incubated at room temperature for 1 h. The sections were then rinsed in phosphate-bufferes saline and incubated at room temperature with EnVision + for 1 h. The antigen-antibody complex was visualized with 3, 3′-diaminobenzidine (DAB) solution [1 mM DAB, 50 mM Tris–HCl buffer (pH 7.69, and 0.006 % H_2_O_2_]. The monoclonal antibodies for AR (AR441) and ERα (NCL-ER-6 F11) were purchased from Dako (Dako, Carpinteria, CA, USA) and Novo-castra Laboratories (Newcastle upon Tyne, UK), respectively. A polyclonal antibody specific for ERβ was raised in rabbits against peptides synthesized to correspond to the C-terminal region of ERβ (CSPAEDSKSKEGSQNPQSQ) [9].

#### Immunohistochemical assessment

The labeling index (LI) was determined by counting the percentage of cells with positive immunoreactivity per 1000 cells [18]. Two trained pathologists (TF and YY) independently evaluated the tissue sections, and the average LI was used. We defined positive immunoreactivity as showing moderate or strong immunoreactivity.

### Statistical analyses

Correlations between age, pretreatment serum PSA levels, J-CAPRA score [15], and LI were evaluated using the Wilcoxon rank sum test. Associations between the group and clinical parameters including Gleason score (GS) and clinical stage were assessed using chi-square tests. BCR-free survival curves were plotted using the Kaplan-Meier method and verified using the log-rank test and univariate Cox proportional hazards regression models. JMP 11.0.0 software (SAS Institute, Cary, NC, USA) was used for all statistical analyses, and *p* < 0.05 was considered to indicate statistical significance.

## Results

### Patient characteristics

**Fig. 2:**
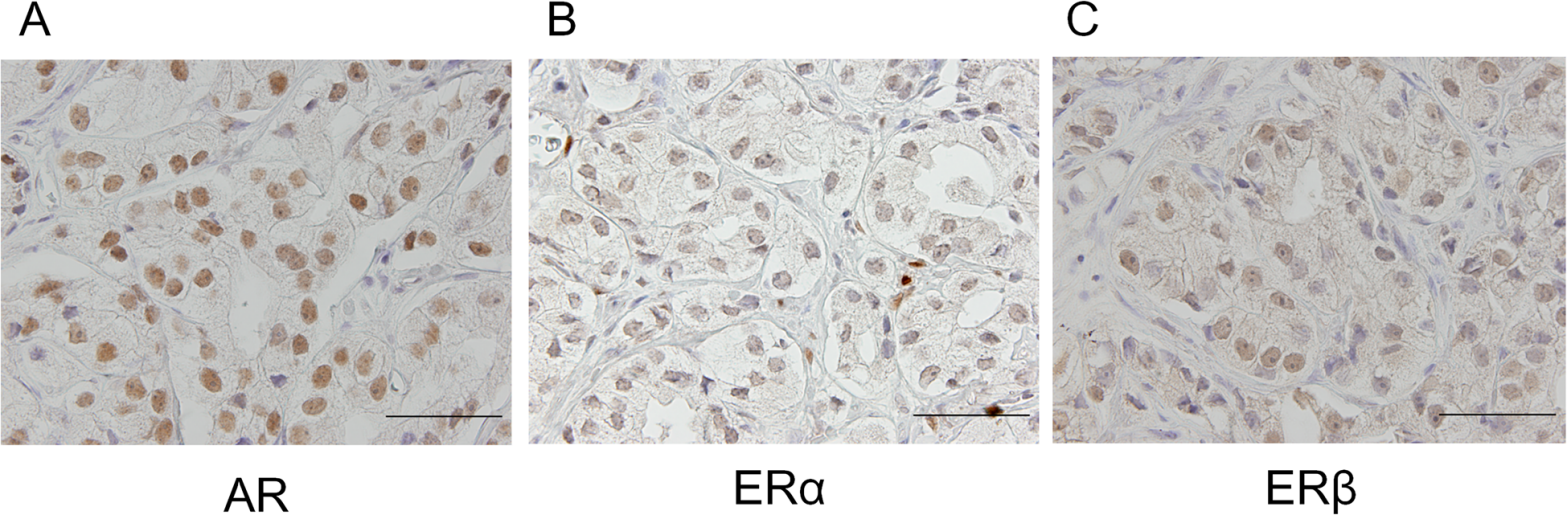
Immunohistochemical staining for AR (**a**), ERα (**b**) and ERβ (**c**) in the tissue sections from the same area of a patient with PC. Strong (**a**) or moderate (**b** and **c**) staining was identified in the nuclei of cancer cells. The LI of AR (**a**), ERα (**b**) and ERβ (**c**) in cancer cells was 100, 35.4 and 26.4, respectively. Scale bar =100 μm

From August 14, 2008 to December 27, 2012, 15 patients were enrolled and randomly allocated to either of the three treatment groups as shown in Table 1. The median age was 74 years (range, 63–85). Pretreatment serum PSA levels were 30–8428 ng/mL (median, 198 ng/mL). The biopsy samples were evaluated by two pathologists as GS 7 (*n* = 3), 8 (*n* = 5), 9 (*n* = 5), or 10 (*n* = 2). The median J-CAPRA score was 9 (range, 6–11). There was no statistically significant difference in age, serum PSA level, stage, GS, EOD, J-CAPRA score or LI against the anti-AR, −ERα, and-ERβ antibodies among the three groups [ADT v.s. TOPADT and ADT v.s. RAPADT; Fig. 2].

**Table 1:** Baseline characteristics (*n* = 15)

		ADT (*n* = 5)	TOPADT (*n* = 5)	*P* value (vs. ADT)	RAPADT (*n* = 5)	*P* value (vs. ADT)
Age (median, range)		76 (74–85)	73 (63–81)	0.59	72 (67–75)	0.29
PSA (ng/mL) (median, range)		223 (30.6–8428)	264 (30–818)	1	126 (30.8–3600)	0.83
Gleason score	7	1	0	0.72	2	0.57
	8	2	2		1	
	9	1	2		2	
	10	1	1		0	
Clinical T stage	2c	2	4	0.19	2	0.55
	3a	3	1		2	
	3b	0	0		0	
	4	0	0		1	
Clinical N stage	0	2	4	0.19	1	1
	1	3	1		4	
Clinical M stage	1b	5	4	1	5	1
	1c	0	1: lung		0	
Extent of disease	1	4	3	0.49	4	1
	2	0	2		1	
	3	1	0		0	
J-CAPRA score (median, range)		9 (8–10)	8 (7–9)	0.37	9 (6–11)	0.92
Labeling index (median, range)						
AR		78.5 (54.8–100)	87.1 (30–100)	1	100 (37.5–100)	0.68
ERα		27.9 (0–46.5)	19.7 (0–37.4)	0.34	20.6 (13.4–35.4)	0.54
ERβ		20.3 (2.4–44.9)	11.9 (7.4–91.4)	0.75	15.2 (7–26.4)	0.9

*ADT* androgen deprivation therapy including surgical or medical castration plus bicalutamide, *TOPADT* toremifene plus ADT, *RAPADT* raloxifene plus ADT, *J-CAPRA score* Japan Cancer of the Prostate Risk Assessment (J-CAPRA) score (ranging from 0 to 12) was calculated on the basis of PSA, Gleason score and clinical stage [15]. Labeling index was determined by counting the percentage of cells with positive immunoreactivity in 1000 cells [18], *AR* androgen receptor, *ER* estrogen receptor

### Primary endpoint

**Fig. 3:**
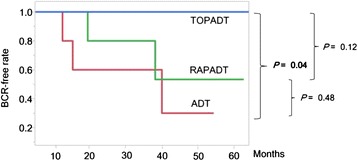
PSA relapse-free survival rates in men with TOPADT, RAPADT and ADT alone (*n* = 15). The PSA relapse-free survival rate in men treated with TOPADT was significantly greater than in men treated with ADT alone (*p* = 0.04)

Table 2 shows the PSA response and outcome after ADT with or without SERMs. One patient discontinued toremifene becauseof a headache during the median follow-up period of 1370 days (range, 431–1983). Five (33 %) (2, 2, 1 in the three groups, respectively) patients achieved a PSA-nadir ≤0.01. At the end of the follow-up period, 5 (33 %) patients (3 in the ADT group and 2 in the RAPADT group) experienced BCR and were switched from bicaltamide to flutamide. One patient in the ADT group became hormone-refractory rapidly and died of PC on day 431 without chemotherapy. One patient in the TOPADT group died of gastric cancer without showing BCR on day 1371. The BCR-free survival rate was significantly higher in men treated with TOPADT than in those received ADT only (*p* = 0.04, ADT vs. TOPADT; *p* = 0.48, ADT vs. RAPADT;, and *p* = 0.12, TOPADT vs. RAPADT; Fig. 3).

**Table 2:** PSA response and outcome after ADT with or without selective estrogen receptor modulators (*n* = 15)

		ADT (*n* = 5)	TOPADT (*n* = 5)	RAPADT (*n* = 5)
Follow-up period (median, range)		1169 (431―1631)	1653 (730―1983)	1570 (750―1883)
PSA nadir (ng/mL)	≥1	1	0	1
	0.01–1.0	2	3	3
	≤0.01	2	2	1
Biochemical recurrence	No	2	5	3
	Yes	3	0	2
Outcome	Alive with disease	4	4	5
	Died of PC	1	0	0
	Died of other disease	0	1: Died of gastric cancer	0
Adverse event		Hot flush: 2	Hot flush: 3, Headache: 1	Hot flush: 3

*ADT* androgen deprivation therapy including surgical or medical castration plus bicalutamide, *TOPADT* toremifene plus ADT, *RAPADT* raloxifene plus ADT, *PC* prostate cancer

Table 3 shows the results obtained from univariate Cox proportional hazards regression models for BCR associated with treatment and the clinicopathological characteristics of the patients, including age, serum PSA levels, J-CAPRA score and LI of AR, ERα and ERβ. TOPADT was only found to be significant in the univariate analysis (*p* = 0.023; hazard ratio, 1.1 e^−9^).

**Table 3:** Univariate Cox proportional hazard regression models of biochemical recurrence free survival (*n* = 15)

	Univariate		
Variable	Hazard ratio	95 % index	*p* value
Age (≥74 vs. <74)	3.9	0.57–75	0.18
Serum PSA (ng/mL) (≥198 vs. <198)	1.9	0.33–15	0.45
J-CAPRA (≥9 vs. ≤8)	1.7	0.27–12	0.57
AR LI (≥93.5 vs. <93.5)	1.2	0.19–8.7	0.87
ERα LI (≥23.2 vs. <23.2)	0.40	0.05–2.5	0.32
ERβ LI (≥16.1 vs. <16.1)	0.73	0.09–4.4	0.73
TOPADT vs. MAB	1.1 e^−9^	0.64–0.64	0.023
RAPADT vs. MAB	0.5	0.069–3.2	0.48

Hot flush was observed in all groups; ADT (*n* = 2), TOPADT (*n* = 3), and RAPADT (*n* = 3); although, all patients continued on therapy.

### Secondary endpoint

**Fig. 4:**
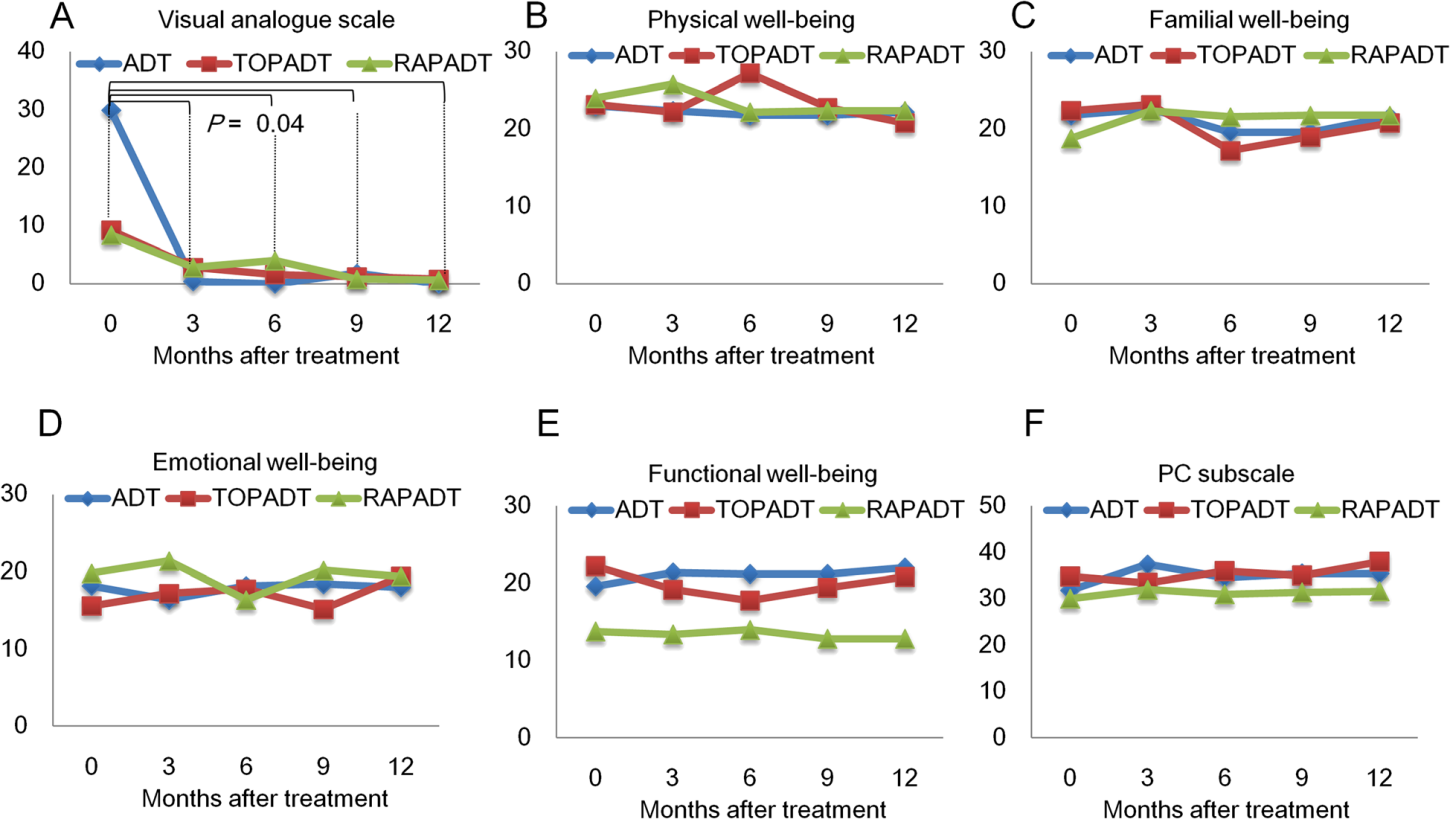
The change in VAS scores following treatment for PC (**a**). The VAS scores were significantly decreased after the treatment (*p* = 0.04, pre treatment vs. 3, 6, 9 and 12 months of treatment). Statistically significant differences were not detected among the three groups. The FACT of before treatment and after 3, 6, 9 and 12 months of treatment. Physical well-being (PWB; **b**), social well-being (SWB; **c**), emotional well-being (EWB; **d**), functional well-being (FWB; **e**) and prostate cancer (PC) scale scores (**f**) were stable during the follow-up period. Statistically significant differences were not detected among the three groups in PWB (*p* = 0.5, pre treatment v.s. 3, 6, 9 and 12 months of treatment), SWB (*p* = 0.5, pretreatment vs. 3, 6, 9 and 12 months of treatment), EWB (*p* = 0.75, pre treatment v.s. 3, 6, 9 and 12 months of treatment), FWB (*p* = 0. 5, pretreatment vs. 3, 6, 9 and 12 months of treatment), and PC sub scale (*p* = 0.25, pretreatment v.s. 3, 6, 9 and 12 months of treatment)

The VAS scores were significantly decreased after treatment (*p* = 0.04, pre-treatment vs. 3 months thereafter), and showed no statistical differences among the three groups (Fig. 4a). Scores of physical well-being, social well-being, emotional well-being, functional well-being, as well as PC subscale scores of FACT questionnaire, were stable during the follow-up period and not statistically different among the three groups (Fig. 4b–f).

## Discussion

The most common initial therapy for metastatic PC is ADT; however, the durability of ADT is limited and affected by various factors including pretreatment PSA level, GS, tumor stage and PSA nadir [19]. The durability of ADT is also influenced by the ER status of the tumor [18, 20].

In fact, estrogens were initially used as one of the earliest forms of treatment agents; however, they were associated with thromboembolic and cardiovascular side effects [21]. SERMs are synthetic estrogen ligands that can exhibit either estrogenic or anti-estrogenic effects depending on tissue types [22]. Toremifene significantly reduced the incidence of PC in a transgenic adenocarcinoma mouse prostate model [23], as well as in men with HGPIN [11]. In addition, toremifene increased the bone mineral density of the hip and spine [24] and improved lipid profiles in men receiving ADT for PC [25]. Raloxifene, which acts as an ER agonist in the bone tissue [26], has been developed for the treatment of osteoporosis in women [27] and showed some tumor-inhibitory effects in CRPC in a pilot study [13]. To date, the anti-cancer effects of these SERMs have not yet been fully investigated in treatment-naïve PC patients. We hypothesized that concurrent use of SERMs would prolong the duration of efficacy of ADT in men with bone metastatic PC.

Currently, we have demonstrated that TOPADT significantly improved the biochemical recurrence rate in men with bone metastatic PC compared with men treated with ADT alone. The results of a recent study showed that the 5-year BCR-free rate was 30 % in men who received ADT plus docetaxel with median serum PSA levels of 26.7 (range, 5.0–106) [5]. In our study, similar rates were noted for men treated with ADT alone (30 %). Surprisingly, the 5-year BCR-free rate in the TOPADT group was 100 %.

Theoretically, the tumor inhibitory effects of toremifene would be mediated via the suppression of ERα-related signals [28, 10]. ERα expression in PC cells was confirmed by immunohistochemistry and quantitative reverse transcription polymerase chain reaction analyses [8, 9, 18]. The mRNA expression of ERα was much lower than AR in PC cells (1:100 ratio); however, ERα expression in cancer-associated stromal cells was significantly related to cancer-specific survival in men with bone metastatic PC [18]. ERα expression was negatively correlated with survival after radical prostatectomy in locally advanced PC [29]. Additionally, ERα promoted proliferation by regulating *MYC* expression and glucose sensitivity in phosphatase and tensin homolog (PTEN)-deficient mouse PC cells [8]. Conversely, depletion of *ERα* inhibited growth in PTEN-deficient mice via a reduction in *MYC* protein and alteration of glucose sensitivity [8]. The results of present study demonstrated that toremifene significantly improved the durability of ADT, suggesting blockade of ERα signaling as a potential target for advanced PC.

ERβ signaling has been associated with a tumor-inhibitory effect in PC through both the classical (ERβ and estrogen-response element complex) and non-classical pathways (ERβ, Krüppel-like zinc finger transcription factor 5, and adenosine 3′,5′-monophosphate response element-binding protein-binding protein complex) [30]. ERβ modulators are expected to inhibit PC growth. Raloxifene exhibits diverse activities via ER depending on whether ERα or ERβ is expressed in the target organ [26]. The results of the present study did not prove a distinct tumor-inhibitory effect mediated by RAPADT as compared to ADT alone. The difference in the reason tumor inhibitory effect between TOPADT and RAPADT may have been attributed to the potency of the drugs and the pattern of ER expression in PC cells. The tumor-inhibitory effect of fulvestrant, another ERβ modulator, was limited because the median time to progression was only 4.3 months in men with CRPC treated with fulvestrant [31]. Further investigations of additional ERβ modulators are warranted with respect to their potential role in the inhibition of human PC.

The known adverse events associated with the use of SERMs include hot flushes, sweating, nausea, dizziness, edema, vomiting and thrombosis [27]. In the present study, two men in the ADT group, two men in the TOPADT group, and three men in the RAPADT group complained of mild hot flushes; however, no medical intervention was deemed necessary. Only one man in the TOPADT group discontinued toremifene administration because of a headache. No events of liver dysfunction or thrombosis were observed.

The present study was not without limitations. The sample size was small, and the cohort was limited to a single institution in an all Asian population. A multicenter external validation study would be necessary to further elucidate the additional effect of toremifene on ADT that we found in patients with advanced PC.

## Conclusions

Results from the present study we demonstrated the good clinical efficacy and tolerability of TOPADT in patients with treatment-naïve bone metastatic PC. Additional clinical trials with larger cohorts are warranted to confirm our promising phase IIA results.

## Additional file

Additional file 1: Table S1. CONSORT 2010 checklist of information to include when reporting a cluster randomised trial. (DOCX 30 kb)

## Abbreviations

ADT: androgen-deprivation therapy
AR: androgen receptor
BCR: biochemical recurrence
CONSORT: consolidated standards of reporting trials
EOD: extent of disease
ER: estrogen receptor
FACT: functional assessment of cancer therapy
J-CAPRA: Japan Cancer of the Prostate Risk Assessment
LI: Labeling index
PC: prostate cancer
PSA: prostate-specific antigen
PTEN: phosphatase and tensin homolog
RAPADT: raloxifene plus ADT
SERM: selective estrogen receptor modulator
TOPADT: toremifene plus ADT
VAS: visual analogue scale
